# Selenium Nanoparticles: A Comprehensive Examination of Synthesis Techniques and Their Diverse Applications in Medical Research and Toxicology Studies

**DOI:** 10.3390/molecules29040801

**Published:** 2024-02-09

**Authors:** Shobana Sampath, Veena Sunderam, M. Manjusha, Zodwa Dlamini, Ansel Vishal Lawrance

**Affiliations:** 1 Department of Biotechnology, Vel Tech Rangarajan Dr Sagunthala R&D Institute of Science and Technology, Avadi, Chennai 600062, India; 2 Centre for Nano Science and Technology, A.C. Tech Campus, Anna University, Chennai 600025, India; 3 Department of Genetic Engineering, School of Bioengineering, SRM University, Kattankulathur 603203, India; 4 SAMRC Precision Oncology Research Unit (PORU), DSI/NRF SARChI Chair in Precision Oncology and Cancer Prevention (POCP), Pan African Cancer Research Institute (PACRI), University of Pretoria, Hatfield 0028, South Africa; 5 Department of Biotechnology, Sree Sastha Institute of Engineering and Technology, Affiliated to Anna University, Chennai 600123, India

**Keywords:** selenium nanoparticle, bio-synthesis, eco-friendly, biomedical applications, anticancer

## Abstract

Selenium is a trace and necessary micronutrient for human, animal, and microbial health. Many researchers have recently been interested in selenium nanoparticles (SeNPs) due to their biocompatibility, bioavailability, and low toxicity. As a result of their greater bioactivity, selenium nanoparticles are widely employed in a variety of biological applications. Physical, chemical, and biological approaches can all be used to synthesize selenium nanoparticles. Since it uses non-toxic solvents and operates at a suitable temperature, the biological technique is a preferable option. This review article addresses the processes implemented in the synthesis of SeNPs and highlights their medicinal uses, such as the treatment of fungi, bacteria, cancer, and wounds. Furthermore, we discuss the most recent findings on the potential of several biological materials for selenium nanoparticle production. The precursor, extract, process, time, temperature, and other synthesis criteria will be elaborated in conjunction with the product’s physical properties (size, shape, and stability). The synergies of SeNP synthesis via various methods aid future researchers in precisely synthesizing SeNPs and using them in desired applications.

## 1. Introduction

Nanotechnology deals with nano-sized particles (1–100 nm) that have attracted attention due to their magnetic properties. Nanoparticles have many positive attributes, such as small size, surface energy, high surface area, and solubility, and they can attach easily to therapeutic agents and be transported [1,2]. These unique properties of nanoparticles have varied applications in medical and biological fields. Nanomaterials are used to treat, diagnose, monitor, and control physical systems [1,3]. Nanoparticles also have scope in biolabeling, biosensing, antimicrobial treatments, and gene therapy [4]. Diverse nanostructures such as dendrimers, polymers, micelles, and nanoparticles like Au, Ag, Zn, Cu, Se, Fe, Ce, Ti, I, etc. are employed in various biological applications [5,6,7]. One such nanoparticle with unique properties is selenium (Se), which is widely studied. Selenium is non-toxic, and colorless, depending on the oxidation state, and acts as the redox center for several antioxidant enzymes [1,8,9]. Selenium is present in various oxidation states: selenate (+6) and selenite (+4). Among these selenium classes, selenite is a latent composite for microbial activity because of its high toxicity [10].

**Figure 1:**
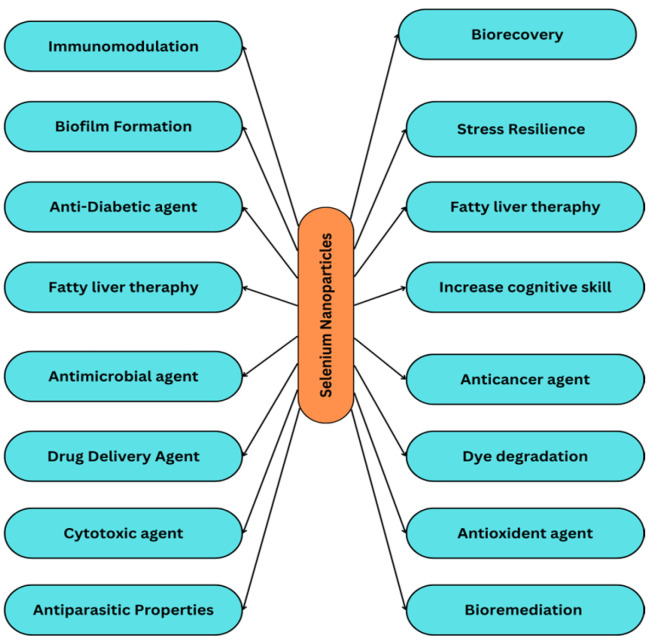
Advantages of selenium nanoparticles in pharmaceutical and biological applications.

Selenium nanoparticles are reported to have some unique properties that make them necessary in many applications (Figure 1). Selenium nanoparticles are reported to have high biological activity and low toxicity [11]. Selenium is an important nutrient for all living organisms, but as it becomes toxic at higher concentrations, it limits its use. Selenium nanoparticles have been proven to exhibit lower toxicity and sub-chronic toxicities than other forms of selenium [12]. This statement has also been proven: selenium nanoparticles have less toxicity than selenomethionine [13]. Comparing Se nanoparticles and Se (IV) nanoparticles at similar concentrations, Se nanoparticles have shown better selectivity between normal and cancer cells [14]. Se nanoparticles have a potent antioxidant property in comparison to selenium species, which makes them a potent compound [15].

Until now, reviews of selenium nanoparticles have focused primarily on their characteristics, production, and applications. In this review, we have discussed biologically synthesizing selenium nanoparticles using bacteria, fungi, and plants. Furthermore, this article elaborates on the various applications of SeNPs in the biological arena, which includes antioxidant, antibacterial, anticancer, wound healing, scolicidal, and anti-viral properties. This review also presents the shortcomings of past work and future aspects of selenium. Since reports on SeNPs are limited, this review will serve as a basis for those who work on SeNPs.

## 2. Methods of Synthesizing Selenium Nanoparticles

**Figure 2:**
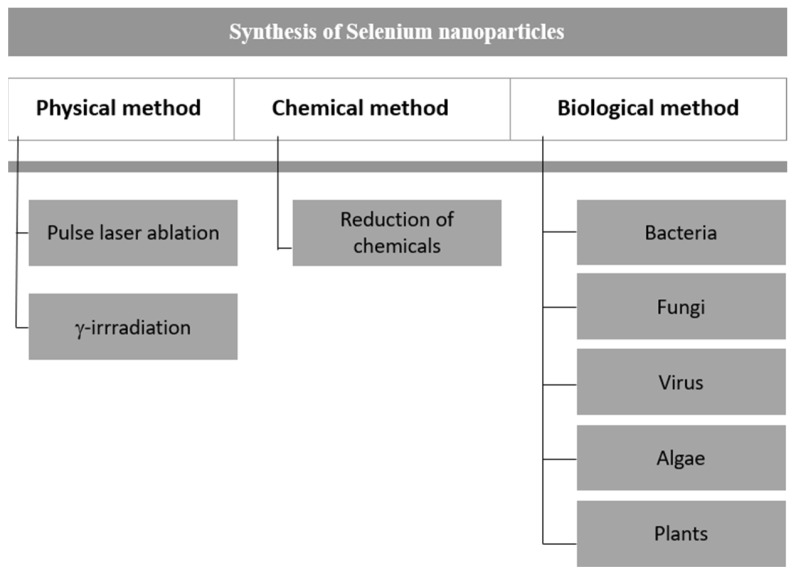
Various methods of synthesizing selenium nanoparticles.

Since the utility of nanoparticles is dependent on their size, shape, and other properties, it is wise to choose the synthesis method according to the study. There are many ways of synthesizing nanoparticles, which are broadly segregated into three main methods: physical, chemical, and biological (Figure 2). We report the synthesis method for synthesizing SeNPs in detail to understand their properties.

### 2.1. Physical Method

In the physical method for making SeNPs, technologies like pulsed laser ablation, vapor deposition, hydrothermal, and solvothermal were used. As it is easy to collect NPs by centrifuging, and because they are very stable, pulsed laser ablation is better than other methods [16]. Table 1 summarizes the different physical methods of synthesizing Se nanoparticles.

Se that is insoluble in water is chosen as a target and immersed in a cell with de-ionized water, as reported by Overschelde et al. It is then irradiated by an excimer laser at a 248 nm wavelength. This is further repeated for 50 min at 50 Hz, and a magnetic stirrer is also fixed to the cell side to avoid beam interaction. This mixed solution was centrifuged at 600 rpm for 25 min to separate the nanoparticles that were characterized, which confirmed the particles were 60 nm in size [17]. SeNPs were synthesized successfully using pulsed laser ablation in liquids without any residual chemicals. A titanium sapphire pulsed laser of 100 fs with a rate of 80 ± 1 MHz was used to irradiate the samples. The rays were directed from the top into a square cuvette that has several selenium pellets at the bottom and 2 mL of de-ionized water. The nanoparticles produced were spherical-shaped and 50–400 nm in size; however, at 60 min, the average size was around 128 ± 30 nm. The synthesized nanoparticles were employed for the study against Candida albicans biofilm [18]. Tzeng et al. have reported the synthesis of both crystalline and amorphous selenium nanoparticles using a femtosecond laser-induced plasma shock wave. A Bl_2_Se_3_ single crystal was irradiated using the laser at a wavelength of 800 nm and a pulse energy of 49 nJ. The nanoparticles formed concentric rings and their sizes, which ranged from 100 to 900 nm [19], defined them. SeCl_4_ was chosen as a precursor and dissolved in distilled water with SDS as a surfactant. It was added under stirring at room temperature and microwaved with 2 mL of hydrazine. After the solution was irradiated at 750 W, it turned a red color, which confirmed the presence of Se^2+^. With further irradiation, the color turned dark red, and the precipitate was cooled, filtered, and washed to obtain nanoparticles that were dried in a vacuum at 70 °C. TEM confirmed that the obtained nanoparticles were sphere-shaped and ranged in size from 5 to 25 nm. The nanoparticles were further fabricated for solar cell applications [20]. An experiment on pulsed Nd: YAG (neodymium-doped yttrium aluminium garnet) lasers for the synthesis of Se nanoparticles was reported by Quintana et al. Amorphous selenium was pressed, and the ablated material was deposited on three substrates: Si wafers, gold-coated glass, and glass microscopic slides. Target (amorphous selenium) and substrate (metallic gold films, silicon wafers, and glass) were maintained in a vacuum chamber with a diffusion pump that gives pressure, and the Se nanoparticles produced were characterized using AFM, which shows the Se nanoparticles from the glass microscopic slide were 90 nm [21]. Selenium quantum dots that were dispersed in double-distilled water were prepared by laser irradiation. At the focal length of a quartz lens, a pulsed Nd: YAG laser with 20 mJ/pulse energy, 10 ns pulse width, and a 10 Hz repetition rate was sent to the solution column. This solution was placed on the copper grid and examined for quantum dots through TEM, which confirmed the particles were 3 nm in size.

### 2.2. Chemical Method

The chemical method for the reduction in inorganic selenium into precursors is the most widely used approach for producing SeNPs. Ascorbic acid, glutathione, cysteine, sodium meta sulfite, and ionic liquid 1-ethyl-3-methylimidazolium thiocyanate, glucose, and fructose were used as reducing agents, while bovine serum albumin, carboxymethyl cellulose, and water-soluble polymers were used as stabilizing agents to prevent nanoparticle aggregation. However, some of these compounds remain, which limits the pharmacological and medical applications of the SeNPs that are made [16]. Table 2 summarizes the Se nanoparticles that were produced by the chemical synthesis method.

Huang et al. described the synthesis of different sizes of nano-selenium to analyze free radical scavenging. Sodium selenite was mixed with GSH containing 200 mg, 20 mg, and 2 mg of BSA which was used for the preparation of different-sized nanoparticles. On adding sodium hydroxide, red Se and oxidized glutathione were formed; they are dialyzed to separate Se, and the final solution is lyophilized and stored. They confirmed the size of the nanoparticle using TEM, where small (5–15 nm), medium (20–60 nm), and large (80–200 nm)-sized nano-Se were obtained [23]. Transferrin (Tf)-selenium nanoparticles were synthesized to describe the cancer-targeted drug delivery system. They prepared fresh solutions of ascorbic acid, selenium dioxide, transferrin, EDC, and chitosan. For the preparation of Tf-SeNPs, selenium dioxide was mixed with DMSO and chitosan solutions, then this mixture was added to the ascorbic acid solution, and finally, after 12 h, the solution was dialyzed. With this, Tf-EDC solutions were mixed and again dialyzed to obtain Tf-SeNPs, which were loaded with Coumarin and used as a probe to determine Tf-SeNP uptake and localization. These synthesized nanoparticles were characterized for TEM, which confirms the nanoparticles were spherical and were stable at 130 nm in size for 21 days without aggregation in aqueous solution, which makes possible their application in the medical field [24].

Huang et al. conducted a detailed study on the antibacterial activity of Se nanoparticles by preparing a selenium nanocomposite. This nanocomposite was synthesized by conjugating quercetin, which exhibits biological activity, and acetylcholine, a neurotransmitter, to the surface of SeNPs. As reported by the authors, SeNPs were prepared by the reduction in Na_2_SeO_3_ by NaBH_4_, then these nanoparticles were capped with quercetin (Qu) and acetylcholine (Ach), finally resulting in Ach-capped SeNPs, Qu-capped SeNPs, and Qu-Ach-capped Se. These nanoparticles were confirmed by the characterization studies. TEM results showed that Qu-capped SeNPs, Ach-capped SeNPs, and Qu-Ach-capped SeNPs were 80–90 nm, 53–68 nm, and 80–90 nm in size, respectively. These nanoparticles were employed as antibacterial agents, and their mechanism was studied by them [25].

SeNPs have been reported to reduce the growth of Staphylococcus bacteria when a colloidal-based synthesis method was employed for the selenium nanoparticle synthesis. The authors have stated the reduction in selenium nanoparticles from sodium selenite and Bovine Serum Albumin (BSA). This reactant solution was reduced using NaOH which serves as a reducing agent, with the formation of selenium nanoparticles noticed by a visible color change to the red solution. The collected nanoparticles were further characterized using TEM, confirming the spherical-shaped nanoparticles with a 40–60 nm size [26]. Polyethylene glycol-selenium nanoparticles were synthesized from gray selenium, a widely available form of Se. The amphoteric property of polyethylene glycol and the coordination among oxygen and selenium atoms lead to PEG-Se nanoparticles [28]. As reported by Kong et al., the SeNPs were known to exhibit pro-cancer potential on prostate LNCaP cancer cell lines. The SeNPs were synthesized from sodium selenite and a glutathione solution containing BSA, to which 1.0 M NaOH was mixed to regulate the pH to 7.1. The change in solution color to red was noticed, confirming the presence of SeNPs.

This was further characterized using DLS, TEM, and XPS, confirming the presence of SeNPs with sizes ranging from 10 to 30 nm. The authors employed these synthesized SeNPs on anticancer activity against prostate cancer cells that suppressed the cell growth partially by caspase-mediated apoptosis [30]. Li et al. used a self-assembly technique to functionalize selenium nanoparticles with 11-mercapto-1-undecanol. Initially, Na_2_SeO_3_ with 0.1 M was prepared, and a vitamin C solution of 50 mM concentration was added dropwise. After completion of the addition, the solution was kept under continuous stirring until a change in color of the solution to red was observed. The pH of the solution was therefore adjusted to 10 by adding NaOH, followed by 11-mercapto-1-undecanol powder that changed the solution color to gray, from which the nanoparticles were collected by centrifugation and freeze-dried. The characterization study reports of the sample revealed that the particle size was 50 nm and that the particles were spherical by TEM and EDX spectroscopy, revealing the presence of chemicals that arrived with the chemical formula Se-capped 11-mercapto-1-undecanol (MUN). The synthesized nanoparticle was studied further for its antioxidant activity and inhibition of cytotoxicity induced by cisplatin, a potent chemotherapeutic drug [31]. Li et al. have synthesized Trolox, which stands for 6-hydroxy-2,5,7,8-tetramethylchroman-2-carboxylic acid, with surface-functionalized selenium nanoparticles by mixing 0.5 g of Trolox with cystamine dihydrochloride of 0.11 g, BOP of 0.39 g, HOBt of 0.134 g, and 0.108 g of DMAP in dry DMF of 10 mL quantity, stirring at R.T. The solvent was washed with sulfuric acid, Na_2_CO_3_, and brine. The final residue was separated by chromatography using silica gel for the purification of Trolox-S. This was then used to produce Trolox-SH functionalized SeNPs by mixing vitamin C solution and Na_2_SeO_3_, and the mixed solution turned red when the pH was adjusted with NaOH. To the amount of 0.1M, 3.86 mg of Trolox-SH powder was dissolved under magnetic stirring, which turned the solution gray. The Se-capped Trolox was characterized as having a wavelength of 220 nm to 100 nm [32]. Wen Liu et al. have prepared a stock solution of 5 mM Na_2_SeO_3_ with sodium selenite powder in 10 milliliters of water. An ascorbic acid solution of 5 mL was added dropwise to the final 25 mL volume of milli-Q water. Then, the solution was stirred at room temperature for 24 h, and the excess 5-FU and sodium selenite were removed by dialysis. The synthesized SeNP was characterized by TEM as being spherically shaped with a 70 nm size. Thus, the authors suggest that 5-FU acts as a key factor in regulating the size of SeNPs [33]. Well-distributed stable selenium nanoparticles were synthesized by Mehta et al. by using two different polarity surfactants: sodium bis(2-ethylhexyl) sulfosuccinate (AOT), which was anionic, and hexadecyltrimethylammonium bromide (CTAB), which was cationic. Initially, they dissolved AOT by stirring in distilled water, to which 5 mL of SeO_2_ was added and hydrazine hydrate of 2.0 mM was supplemented in stirring conditions. After a few hours, when the solution turned orange, it was centrifuged, washed with ethanol, and dried in a vacuum desiccator. Then, the particles were characterized using XRD and TEM, where the SeNPs were spherical and had a monoclinic structure with a size of 14–17 nm. FTIR was also used to examine the particles, which show the alignment of deposited surfactant molecules on the surface of selenium. The authors conclude that the specific properties of these SeNPs can be employed in photoelectric devices [36]. The antioxidant capacity of the SeNPs was reported by Zhai et al. which were synthesized and stabilized with CS3 and CS200. The morphology of the nanoparticles was observed using STEM, which confirmed that the CS-SeNPs were 50 nm in size with a spherical shape. The synthesized CS-SeNPs were studied for their antioxidant activity [34]. SeNPs were reported to be reduced from selenitic acid by using the template sodium alginate. Initially, a suitable volume of sodium alginate was combined with a selenious acid solution, to which ascorbic acid was added to start the reaction, which continued for 2 h and was completed by ultrasonication followed by centrifugation, yielding red SeNPs with a size range of 20–70 nm. These nanoparticles were studied for their apoptotic activity on human hepatic cancer cells from Bel7402 [35].

### 2.3. Biological Method

#### 2.3.1. Synthesis of Selenium Nanoparticles Using Plant Extracts

**Figure 3:**
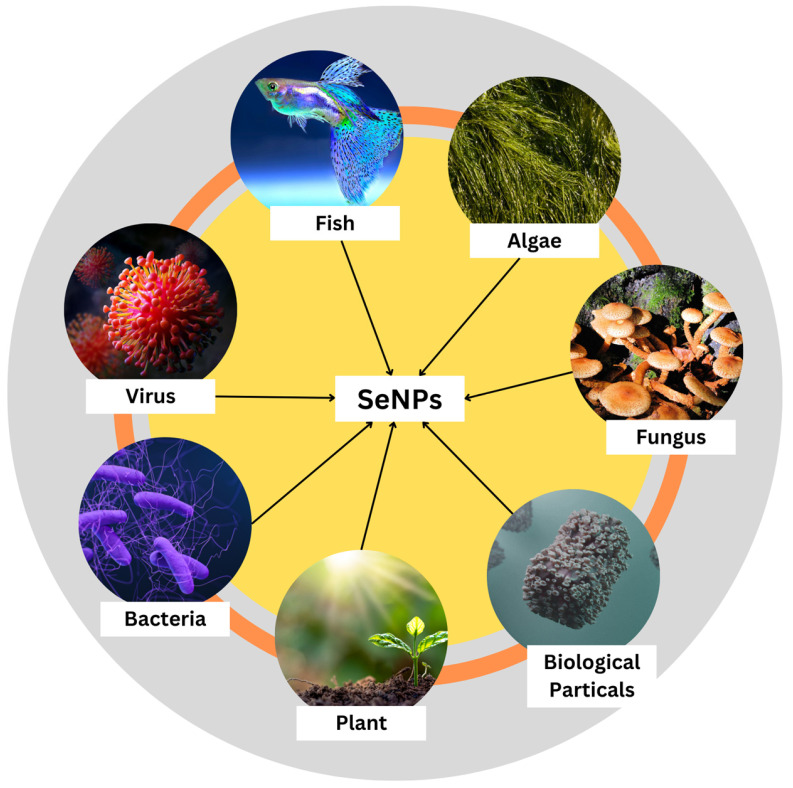
Various modes of biological synthesis of nanoparticles.

SeNPs are synthesized using different biological means such as fungi, viruses, bacteria, plants, algae, and other biological particles such as protein molecules (Figure 3). Plants have already been identified as being useful in the production of SeNPs. Raw materials are readily available, and many of these plants possess traditional and medicinal uses. Agricultural waste materials, including fruit peels, could also be used in the process. Plant materials high in polyphenols, saponins, flavonoids, alkaloids, polysaccharides, and other reducing and stabilizing agents have the best chance of succeeding. The phytochemical screening only found the most important active ingredients in a qualitative way [16]. Sharma et al. have reported a biological molecule-mediated synthesis of selenium nanoparticles as an alternative to the chemical method of synthesis. They used *Vinifera* fruits that were drenched overnight, crushed, and refluxed in distilled water for the extraction of filtrate that was refluxed with selenious acid for 15 min to synthesize SeNPs. They were then centrifuged and the SeNPs were collected. On the characterization of these nanoparticles, the size and morphology of the synthesized selenium nanoballs were 3–18 nm, as confirmed by TEM and FTIR spectrum results, which confirmed the presence of glucose and fructose content in the dried *Vitis vinifera* that acts as a reducing agent. Thus, the authors provided a simple ecofriendly method of synthesis of selenium nanoballs that can be employed for biological applications [37].

Prasad et al. have reported an environmentally friendly synthesis of SeNPs for their protective effect on DNA damage caused by UV. Fresh young leaves of the lemon plant were chosen for the study and cut into small pieces. The leaves were softened in 20 mL of Tris-CL using a mortar pestle. A thick slurry of leaf was taken and centrifugated at 10,000 rpm at 4 °C for 5 min. The supernatant obtained was used as a precursor for the synthesis of Se nanoparticles. The extract was dropwise added against sodium selenite(solution) under stirring which was then rotated in a shaker for 24 h at 200 rpm. The reduction in Se ions was monitored using UV-vis spectra. Characterization studies of the SeNPs using TEM, SEM, CRD and EAX showed the particles were polydisperse, crystalline with sizes ranging from 60–80 nm. The synthesized SeNPs were studied for their protective effect against DNA damage induced by UV radiation [38]. A simple procedure of synthesizing SeNPs from fenugreek seeds was reported by Ramamurthy et al. where they prepared the filtrate by mixing 1% of fenugreek seeds in distilled water for 15 min and the extract was filtered. This filtrate of 1 mL was mixed with a 30 mM selenic acid solution of 40 mM of ascorbic acid. After 24 h incubation, the prepared solution was centrifuged and the pellets were washed, dried, and collected. The collected red SeNPs were suspended in PBS, sonicated, and further centrifuged to obtain the powder form of the pellet. The obtained green synthesized SeNPs were 50–150 nm in size and oval in shape as characterized by SEM. They were employed for the study on cytotoxicity against MCF-7 cells [39]. SeNPs were synthesized from broccoli that was sun-dried and the powder was employed for the synthesis of nanoparticles. An amount of 0.7 g of broccoli powder was selected and then boiled with 70 mL of distilled water, then the obtained extract was filtered from which 30 mL of extract was added to 30 mM of sodium selenite and kept under continuous stirring at 450–600 rpm for 2 to 3 days. Furthermore, the nanoparticles obtained were confirmed through TEM and SEM that revealed the particles were less than 50 nm in size and were studied for their anticancer activity [40]. The two citrus species, i.e., *Citrus paradisi* and *Citrus limon*, were reported as effective reducing agents in the synthesis of SeNPs that hold effective antibacterial activity [41].

#### 2.3.2. Synthesis of Selenium Nanoparticles Using Biological Particles

A simple wet chemical method has been reported by Ingole et al., where SeNPs were synthesized by the reduction of 10^−1^ M of sodium seleno sulphate with 10 mL of 4% glucose solution and the mixture was refluxed. A change in the color of the solution was observed to be yellow, which lasted for months with glucose; but without glucose solution even after prolonged heating, no change in color was found. So, this study proves that glucose acts as the reducing agent to reduce selenious acid to SeNPs. On characterization with UV, XRD, and TEM, SeNPs synthesized were spherical with 20–80 nm size [11]. A first comparative study between chitosan-modified SeNPs and other seleno species—Se (IV), Se (VI), SeMet, SeCys2, Se-MeSeCys on hepatocarcinoma (HepG2) cells—was conducted by Estevez et al. To perform this study, Ch-SeNPs were prepared by mixing the 10 mL of chitosan polysaccharide solution with 7.5 mL of ascorbic acid and 5 mL of acetic acid solutions; to this mixture, sodium selenite (0.25 mL) was slowly added. The formation of SeNPs was confirmed by the change in color of the solution from colorless to red, then the solution obtained was dialyzed for 2 h at R.T. Chitosan added were tested at different concentrations in particle size and TEM analysis confirmed that at 0.1% concentration, nanoparticles were of 40 and 60 nm. Thus, they used a 0.1% concentration of chitosan throughout their experiment [12]. SeNPs were set by reducing the sodium selenite with cysteine solution by dropwise titration under magnetic stirring. The final concentration of the mixture was characterized that confirmed the presence of SeNPs. This study synthesized mono-dispersed and homogeneous spherical SeNPs of 100 nm at a concentration of 4:1 L-cysteine to sodium selenite. Thus, the authors paved the way for the large-scale synthesis of SeNPs with no organic solvents or strong reducing agents required (Table 3) [42].

#### 2.3.3. Synthesis of Selenium Nanoparticles Using Bacteria

Dhanjal et al. have studied SeNPs synthesis from species isolated from coalmine soil that was identified as *Bacillus cereus*. This species was identified to have the ability to change toxic selenite to SeNPs by aerobic detoxification of selenite and also can withstand high toxic concentrations of sodium selenite. A bacterial strain with 2 mM of sodium selenite was incubated at 37 degrees Celsius at 200 rpm for 48 h; they were then centrifuged and the pellets were collected. Furthermore, characterization studies on SeNPs confirm that particles were 150–200 nm in size with a high negative charge (−46.86 mV) and were stable [10]. SeNPs synthesis from *Bacillus* species MSh-1 and its toxicity analysis were studied by Shakibaie et al. Nutrient broth with Se^4+^ ions were mixed for aerobic cultivation and kept for 14 h. Centrifugation was employed to separate the bacterial cells and SeNPs. This study thus proves that *Bacillus* sp. acts as a reducing agent to reduce Se^4+^ ions to SeNPs and the synthesized nanoparticles were 80 to 220 nm in size. The synthesized nanoparticles are employed to study acute and sub-acute toxicity in mice [38]. *Bacillus megaterium* was cultured, centrifuged, and the cell–free supernatant was mixed with 1 mM of selenious acid suspension at a controlled temperature of 25 °C and the color change was noted to be reddish-brown; furthermore, the solution was centrifuged and the pellets were dried and was reported to have effective antifungal activity against *Rhizoctonia solani* [55]. Sodium selenite solution was reduced to SeNPs using glucose, ascorbic acid, and starch solution and they have adopted a dropwise titration method to prevent agglomeration of particles. The synthesized SeNPs were spherical shaped with about 2 to 10 nm and they were reported to have effective antibacterial activity against *Mycobacterium tuberculosis* H37Rv strain and cytotoxic activity on colorectal cancer cell lines [56].

Sweety and her co-authors have worked on the selenium nanoparticles synthesis from *Acinetobacter* sp. SW30, which was isolated by sewage sludge. The precursor employed was sodium selenite that was suspended in the bacteria, where the extraction process was found difficult and therefore, on suspending the particles with phosphate buffer and repeated centrifugation, the clear solution called TCP was obtained from which selenium nanoparticles were synthesized. Amorphous nanospheres of 78 nm were synthesized at 1.5 mM and crystalline nanorods were synthesized at 2.0 mM Na_2_SeO_3_ concentration in an 18 h culture with 2.7109 CFU/mL. In the solution of 4 mg/mL TCP, polygonal-shaped SeNPs with a normal size of 79 nm were produced. The manufactured SeNPs were tested against breast cancer cells in an anticancer investigation [47]. Khiralla and Deeb reported a study on SeNP synthesis from *Bacillus licheniformis* that was isolated from food waste. The bacteria were initially isolated and after the incubation period, it was centrifuged and incubated with 1 mM of selenium dioxide for 48 h at 37 °C. After the incubation period, a visible inspection in the change of color was noticed that confirmed the formation of SeNPs. Characterization study confirmed the SeNPs were spherically shaped with a size range from 10–50 nm. Then, the authors subjected the synthesized SeNPs to antimicrobial, antibiofilm, and toxicity analysis that proved to be very effective in preventing the biofilm formation by the foodborne pathogens [48]. Hossein Mahmoud and his coauthors have synthesized SeNPs from a marine bacterial strain *Bacillus* sp. Msh-1. Initially, a sterile nutrient broth was suspended with Se^4+^ ions. The medium was inoculated with *Bacillus* sp. Msh-1 fresh inoculum was incubated aerobically at 30 °C for 14 h after which the bacterial cells and SeNPs were separated by centrifugation where the synthesized nanoparticles were spherically shaped with size 80–220 nm as confirmed by TEM. These SeNPs were studied for the scolicidal effect against Cystic echinococcosis, a zoonotic parasitic infection [52].

Actinobacterium named *Streptomyces minutiscleroticus* M10A62 was utilized for the synthesis of SeNPs that were isolated from a magnesite mine. Soil samples of the magnesite mine were collected, diluted, and agar plates were amended with nystatin and nalidixic acid to avoid bacterial and fungal formation. A potential strain M10A62 was used for the synthesis of SeNPs. The bacterial strain was mixed with 100 mL of yeast extract and rotated in a shaker for 5 days at about 200 rpm after which the extracted biomass by centrifugation was washed and dissolved in an aqueous solution of 1 mM of sodium selenite and rotated in a shaker for 72 h. The formation of SeNPs was visible by the formation of red color and then the produced nanoparticles were centrifuged and the nanoparticles were obtained. The SeNPs formed were characterized by XRD, TEM, EDX, and FTIR that confirmed the synthesized SeNPs were spherically shaped with 100–250 nm size. The biogenically synthesized SeNPs were studied for their cytotoxic, antibiofilm, wound healing, antioxidant, and anti-viral activities [53].

SeNPs were synthesized from a bacterial strain *Bacillus* sp. Msh-1 which was obtained from the Caspian Sea. To synthesize SeNPs, 100 mL of sterile nutritive broth containing SeO_2_ was seeded with 1 mL of fresh *Bacillus* sp. MSh-1 inoculums and incubated at 30 °C for 14 h in a shaker. The cells were then centrifuged to extract biomass. This was ground in liquid nitrogen and the slurry was ultrasonicated that was washed and centrifuged to obtain the SeNPs that were further examined using different characterization techniques. Based on the micrographs from TEM, the obtained SeNPs were spherical shaped of size ranging from 80–220 nm. Also, the elemental composition from the SeNPs indicated solid signals from the Se atoms confirming the presence of SeNPs. These obtained SeNPs were studied on the biofilms against three bacterial species (*P. mirabilis*, *S. aureus*, *P. aeruginosa*) and their studies were compared to selenium dioxide [54]. SeNPs synthesized from *Zooglea ramigera* were reported by Srivastava et al. The activated *Z. ramigera* and sodium selenite were added together into a 100 mL broth medium that was incubated for 48 h at 30 °C at 150 rpm. The color change from yellow to red was visible evidence of the formation of SeNPs. After the particles were taken from the mixture, it was centrifuged at 12,000 rpm for 10 min and the SeNPs were collected for further characterization studies. The bacterium thus was found to be an efficient source for the formation of SeNPs. The formed SeNPs were spherical with a size of 30 to 150 nm and the SeNPs were reported to be stable for six months with minimal aggregation [40]. *Bacillus amyloliquefaciens*, a novel marine bacterial strain, was employed for SeNPs synthesis where 2.5 mM of selenite stock was mixed to bacterial suspension and kept for 24 h at 150 rpm in a shaker where the formed SeNPs were confirmed using UV-Vis spectroscopy and a noticeable color change was reported by the authors [57].

#### 2.3.4. Synthesis of Selenium Nanoparticles Using Virus

Huang et al. has reported the synthesis of SeNPs using TGS (swine transmissible gastro enteritis virus). Initially, SeNPs were prepared using sodium selenite and BSA by adjusting the pH to 7 using sodium hydroxide. Red selenium formed during this stage was dialyzed and lyophilized [23]. This colloidal selenium is then conjugated with *Alphacoronavirus* (TGA) using antigen solution and hydrazine hydrochloride to 1 M solution of sodium selenite. This drug is studied to investigate the immunogenic properties of SeNPs [58].

#### 2.3.5. Synthesis of Selenium Nanoparticles Using Algae

Size-controlled formation of SeNPs using the polysaccharide of *Undaria pinnatifida* was investigated for the treatment of human melanoma cells. *U. pinnatifida* polysaccharide solution was added with ascorbic acid and to this, sodium selenite solution was added slowly and sonicated. These solutions were dialyzed and determined by ICP-AES analysis. Characterization of these nanoparticles showed that the particles are uniformly spherical with sizes ranging between 44–92 nm. This work by Chen et al. has proved to be a modest method for the synthesis of stable SeNPs for over 3 months, which proves to be an important criterion for medical applications [14]. A solution-phase method of synthesizing SeNPs using Spirulina polysaccharides (SPS) was reported by Yang et al. Spirulina platensis was extracted using hot water and the precipitate was freeze-dried to a brown powder that was further purified with diethylamino ethyl-cellulose ion-exchange chromatography. This precipitate was freeze-dried to form a 98% pure white SPS powder. This prepared SPS was employed to produce selenium nanoparticles by mixing with sodium selenite solution of 2 mM concentration. To this solution, fresh ascorbic acid was added and dialyzed using Milli-Q water until no trace of Se was observed. TEM images of these nanoparticles revealed that particles are aggregated without SPS, whereas with an SPS concentration of 50 mg/L, SeNPs formed a homogeneous spherical structure with 20–50 nm size. As the particle size and stability of the nanoparticle is an important factor for the biological application of the synthesized nanoparticle, this study explained that at different concentrations, nanoparticle size differs and so at 50 mg/L concentration is apt for the SeNPs synthesis that exhibited 48.6 nm particle size. Though particle size decreases at 100 mg/L, the yield of the nanoparticle is affected. Thus, the synthesized nanoparticle was further studied for the anticancer activity on MCF-7, A375, HeLa-229, and MG-63 cancer cells [46].

#### 2.3.6. Synthesis of Selenium Nanoparticles Using Fish

Though SeNPs were synthesized using many biological methods, the synthesis of selenium nanoparticles from the gill of *Labeo rohita* fish was reported by Kumar et al. The extract from the gill was added to 2 M of sodium selenite in 200 mL of distilled water and shaken for 96 h; the final solution was centrifuged at 600 rpm for 15 min and the pellets were dried and crushed into fine powder. The synthesized SeNPs on characterization using particle size analysis confirmed that the particles were 206.6 nm in size. They were employed to determine the toxicity of Se and Se nanoparticles on *Pangasius hypophthalamus* [49].

#### 2.3.7. Synthesis of Selenium Nanoparticles Using Fungi

Zare et al. have synthesized SeNPs from a fungus that was isolated from the soil sample. A fungus named *Aspergillus terreus* was isolated from the soil that was employed for the synthesis of Se nanoparticles. SDB medium was prepared which was inoculated with fresh inoculum and subjected to aerobic incubation for 7 days. Fungal cells were collected from the medium by centrifugation and the supernatant was filtered. This filtrate was added to 80 mL of Se ionic solution that after incubation at room temperature for 60 min formed selenium nanoparticles. The formed SeNPs were isolated by centrifugation and the red sediments were centrifuged and separated. The formed nanoparticles were confirmed with UV-visible spectroscopy, SEM, and EDAX. Thus, the authors conclude that the isolated fungus was able to convert Se^4+^ ions into SeNPs with particle size less than 100 nm which has various biological applications and can also be scaled up for large-scale processes [51].

## 3. Biomedical Applications of Selenium Nanoparticles

**Figure 4:**
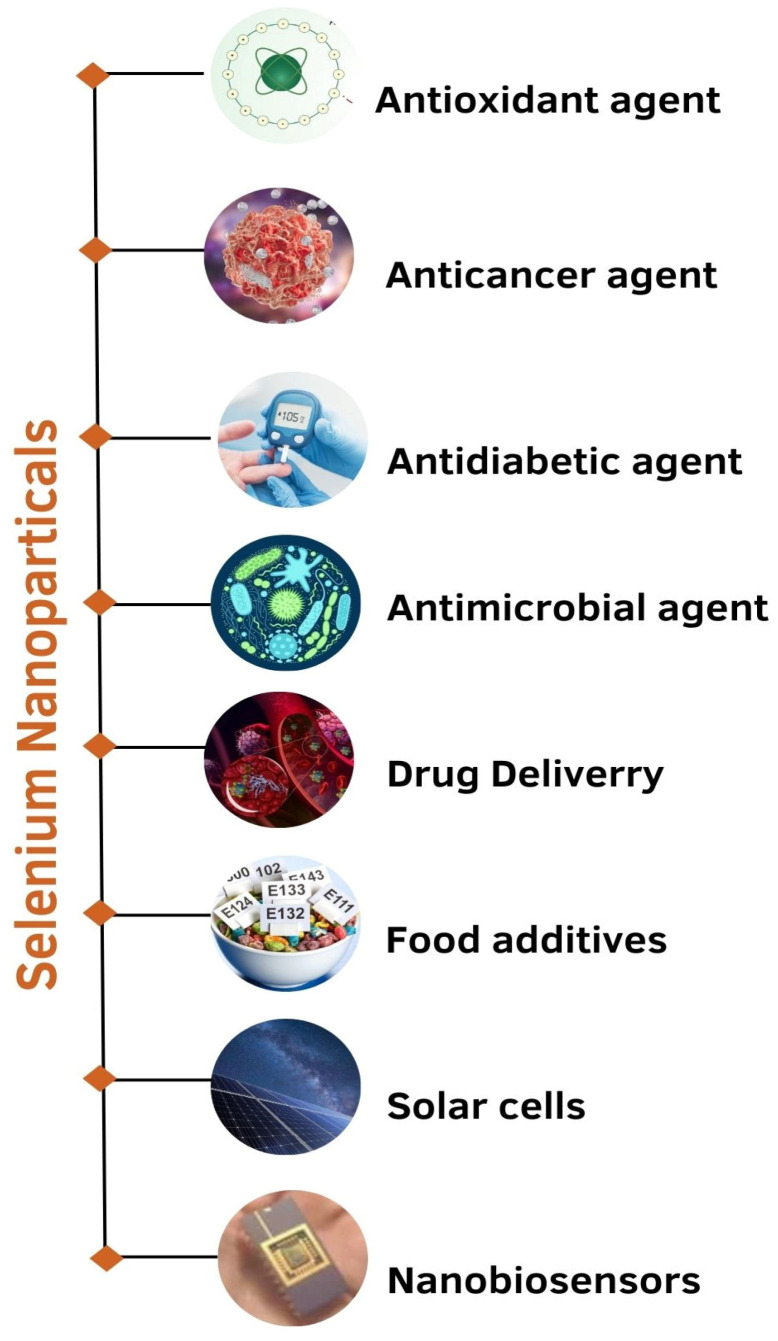
Applications of selenium nanoparticles.

The various applications of Se nanoparticles have been listed below (Figure 4):

### 3.1. Antibacterial Activity of Selenium Nanoparticles

**Figure 5:**
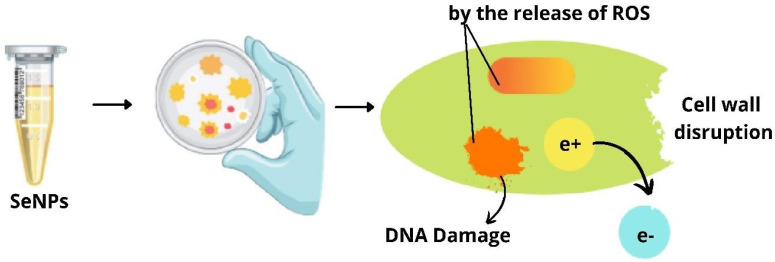
Damage to the bacterial membrane by selenium nanoparticles.

Due to interactions between SeNPs and several functional groups (C–O, C–N, NH, and COO–) of proteins, SeNPs have strong adsorptive and biological activity, allowing them to be used as an antibacterial agent [41]. Selenium-conjugated nanoparticles with quercetin and acetylcholine were synthesized by Huang et al. to determine their effective activity against multidrug-resistant superbugs *Escherichia coli* and *Staphylococcus aureus*. Ach-capped SeNPs, Qu-capped SeNPs, and Qu-Ach-capped SeNPs were synthesized and a comparative study of these nanoparticles on their antibacterial properties was investigated by the authors. Qu-Ach SeNPs have more bactericidal activity on superbugs, owing to the presence of acetylcholine which binds to the bacterial cell membrane leading to the leakage of cytoplasm causing bacterial death as depicted in Figure 5. Thus, these nanoparticles prove to be a potential application for treating infectious diseases [25].

**Figure 6:**
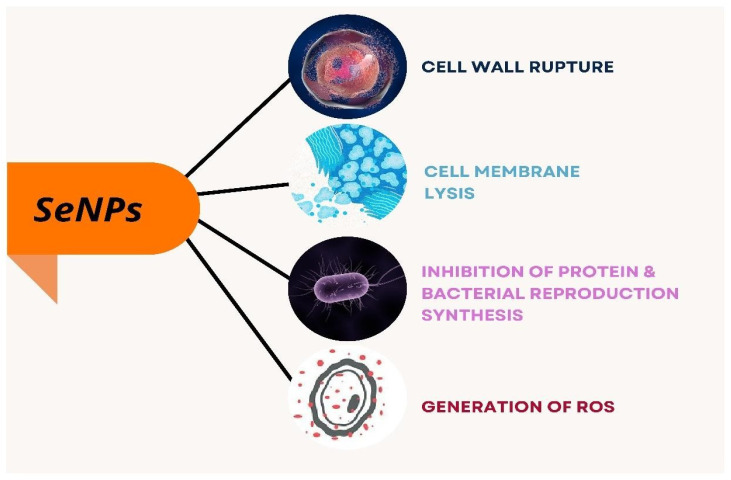
Different modes of action of selenium nanoparticles on bacteria.

Phong et al. have reported on analysis of the nanoparticles on the growth of *Staphylococcus* bacteria that inhibited the growth formation of *S. aureus* by 60 times, preventing biofilm formation [26]. Selenium nanoparticles were synthesized from *Bacillus licheniformis* by Khiralla and Deeb that they employed for antimicrobial activity against six foodborne microbes, *Bacillus cereus*, *Staphylococcus aureus*, *Enterococcus faecalis*, *Escherichia coli*, *Salmonella typhimurium*, and *Salmonella enteritidis*. Though the different concentration of SeNPs was employed for the antibacterial activity, SeNPs at a concentration of 25 µg/mL exhibited a significant effect on all the bacteria chosen for the study. This was supported by their study that *B. cereus* and *E. coli* were resistant to SeNPs at 15 and 10 µg/mL, respectively. Thereby, the authors conclude that bacterially synthesized SeNPs have an adverse effect on the foodborne pathogenic strains [48]. SeNPs act on bacteria through different modes that cause lysis of bacteria leading to cell death as explained in Figure 6, and the penetration of SeNPs to the bacterial membrane causes its damage and results in bacterial lysis as depicted in Figure 5.

### 3.2. Anti-Viral Activity of Selenium Nanoparticles

The actinobacterium *Streptomyces minutiscleroticus* synthesized SeNPs by Ramya et al. have been reported to have potential anti-viral activity against type-1 dengue virus.

**Figure 7:**
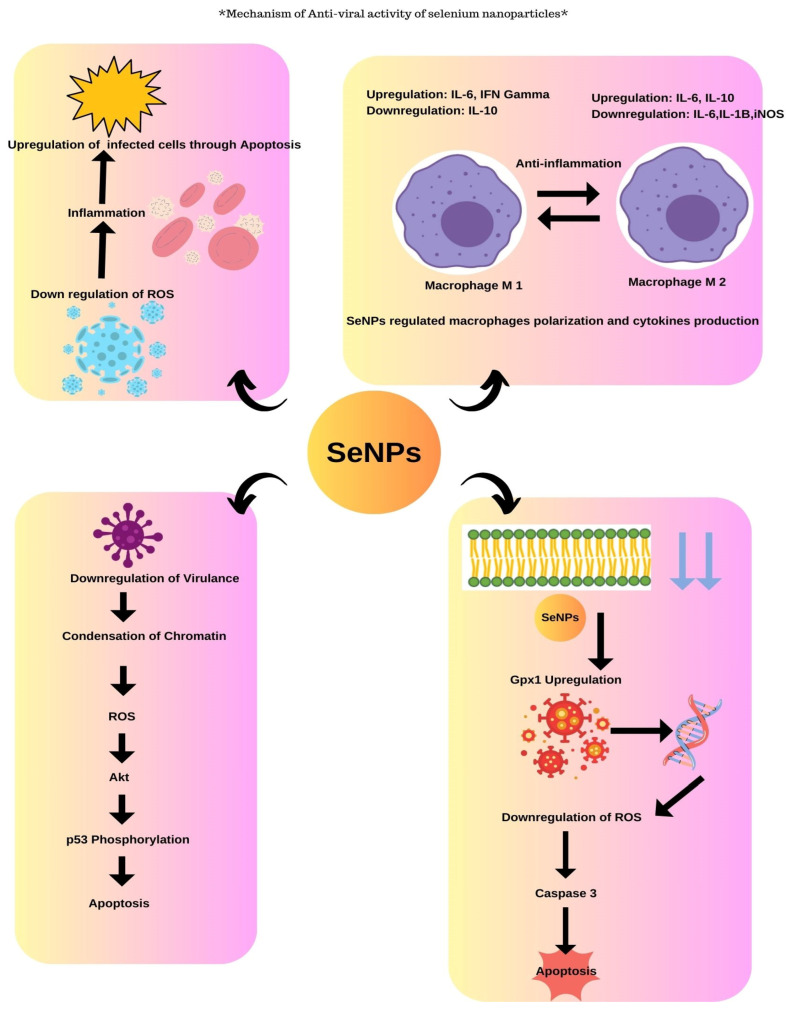
Anti-viral activity of selenium nanoparticles.

SeNPs were known to exhibit anti-viral activity through different mechanisms. SeNPs help in the downregulation of virulence by the chromatin condensation and by the release of ROS causing apoptosis/cell death. The SeNPs exhibit anti-viral activity by the upregulation of GP×1 and downregulation of caspase 3 genes that result in apoptosis (Figure 7). The Vero cells were grown on the 24-well plate where the inhibitory concentration was assessed and about 100 µL of viral suspension was incubated for 1 h and then the viral suspension was added to SeNPs at different concentrations which were incubated for 7 days after which the results were analyzed that exhibited maximum viral growth at 700 ppm; also, the anti-viral activity tended to increase with the increase in dosage as studied by the authors [50].

### 3.3. Use of Selenium Nanoparticles as an Antibiofilm Agent

SeNPs of size 10–50 nm from *Bacillus licheniformis* were studied for the antibiofilm effect at 20 µg/mL concentration against six foodborne pathogens, *Bacillus cereus*, *Enterococcus faecalis*, *Staphylococcus aureus*, *Escherichia coli* O157:H7, *Salmonella typhimurium*, and *Salmonella enteritidis*, using the polystyrene microtiter plate method. The results of their study showed a sharp effect of selenium nanoparticles as an antibiofilm mediator against *Enterococcus faecalis*, *Staphylococcus aureus*, *Salmonella typhimurium*, *Escherichia coli*, and *Salmonella enteritidis* where they lost 100% of their biofilm-forming ability. The synthesized SeNPs were also studied for their biofilm-removing ability but they failed to do so; however, their effect on preventing the biofilm formation was very effective on all studied pathogens except *B. cereus* as biofilm formation is a virulence factor of the foodborne pathogens,; the study conducted by the authors acts as an alternative way to prevent the biofilm formation [48]. Ramya et al. have reported the potential application of actinobacteria-synthesized SeNPs as an antibiofilm agent. The bacterial strains were cultured and inoculated with SeNPs that were considered as the positive control and the broth with SeNPs were taken as the negative control. After an incubation period of 48 h, it was splashed with phosphate saline buffer and the optical density was taken at 570 nm where the results showed that SeNPs inhibited the biofilm formation of actinobacteria species at a very low concentration of 3.2 µg. Thus, the inhibitory action of SeNPs on the biofilm-forming ability of actinobacteria was studied [50].

**Figure 8:**
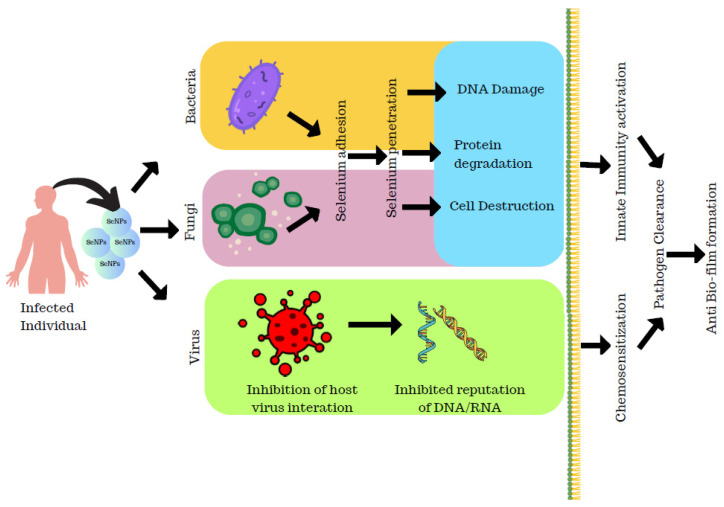
Mechanism of selenium nanoparticles as antibiofilm agent.

Shakibaie et al. reported a study on the action of SeNPs against the biofilm-producing capacity of bacterial strains. Initially, the authors collected samples from patients and isolated around 30 species of *P. mirabilia*, *S. aureus*, and *P. aeruginosa*. The potent biofilm-formed variants were isolated using a biofilm-formation assay. Initially, the antibiotic resistance of each strain was analyzed against commonly used antibiotics and the test results of minimum inhibitory concentration for these strains were resistant to SeNPs and SeO_2_ at 100 µgmL^−1^. Hence, the antibiofilm activity of SeNPs and SeO_2_ was determined at a sub-MIC concentration of SeNPs and SeO_2_. At 2 µgmL^−1^, biofilm formation decreased and maximum reduction was observed at 16 µgmL^−1^ in the case of *S. aureus*. For *P. mirabilis*, the decrease in the biofilm activity was observed at 0–16 µgmL^−1^ which remained constant at 2 µgmL^−1^, whereas for *P. aeruginosa*, the biofilm-forming ability was reduced at 0–16 µg mL^−1^ for both SeNPs and SeO_2_. The authors also conducted a study on the sequel effect of temperature and pH against the formation of biofilms where their results revealed that the biofilm inhibitory effect was higher for SeNPs at 25 °C and 37 °C than SeO_2_ for *S. aureus* and *P. aeruginosa* than on *P. mirabilis*. The effect of pH on SeNPs and SeO_2_ biofilm formation revealed that while the biofilm-forming action of SeNPs on the three strains of bacteria was not considerably different at pH 7 and 9, the biologically active SeNPs represented a considerable rise in biofilm action for *S. aureus* and *P. aeruginosa* should be able to colonize at pH 5 [54]. The SeNPs synthesized using pulsed laser ablation were employed as an antibiofilm agent against *Candida albicans* biofilm. Also, their study confirmed that crystalline nanoparticles also contributed to the biofilm activity of SeNPs. Thus, the SeNPs synthesized using pulsed laser ablation acted as an effective antibiofilm agent against *Candida albicans* biofilms [18] (Figure 8).

### 3.4. Promotion of Immune Response by Selenium Nanoparticles

**Figure 9:**
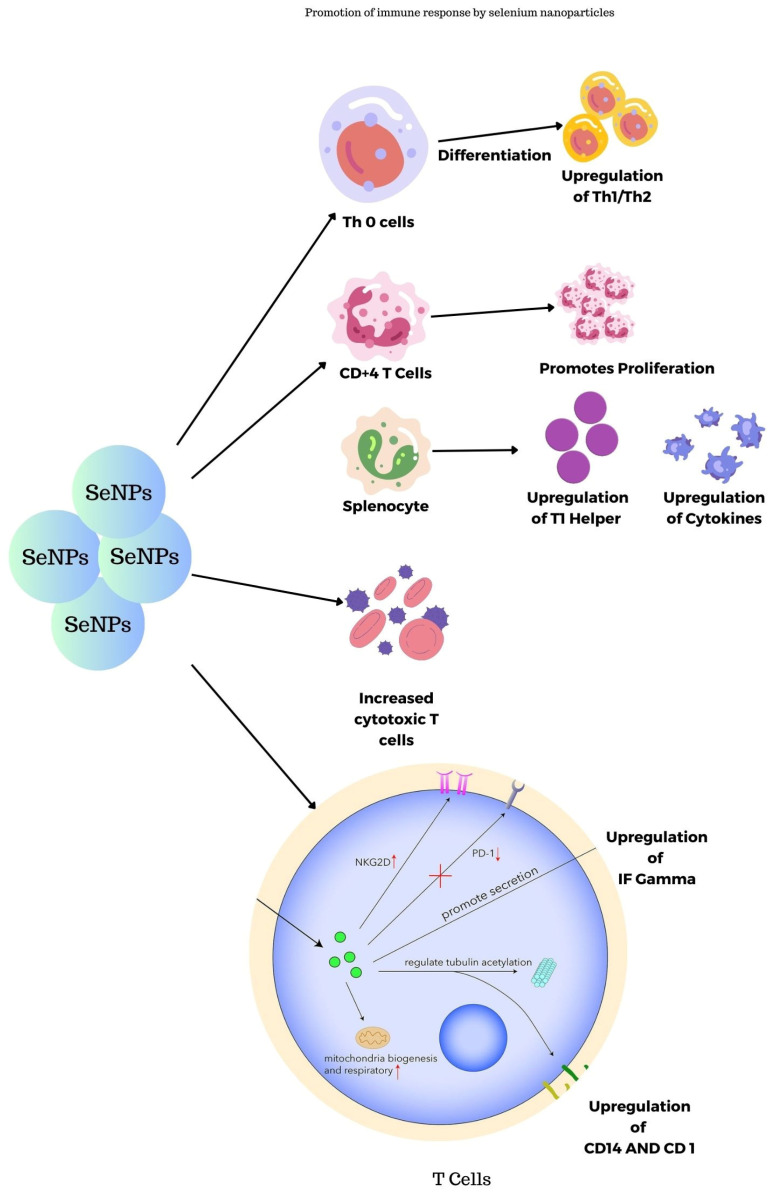
Promotion of immune response in the body by selenium nanoparticles.

SeNPs synthesized using transmissible gastroenteritis virus were studied for their immunogenic properties in guinea pigs as reported by Staroverov et al. The blood plasma collected was investigated and the results show that they lead to the activation of respiratory and peritoneal macrophages thus leading to the production of monoclonal antibodies. In turn, they stimulate the production of interferons and cytokines and also serve as nanocarriers for viral peptides to cytotoxic T-lymphocytes [59]. Figure 9 shows the immune response promotion of SeNPs in the body. SeNPs activate the Th cells, splenocytes, CD^4+^ T cells, and Tc cells, thereby promoting the immune system.

### 3.5. Scolicidal Effect of Selenium Nanoparticles

**Figure 10:**
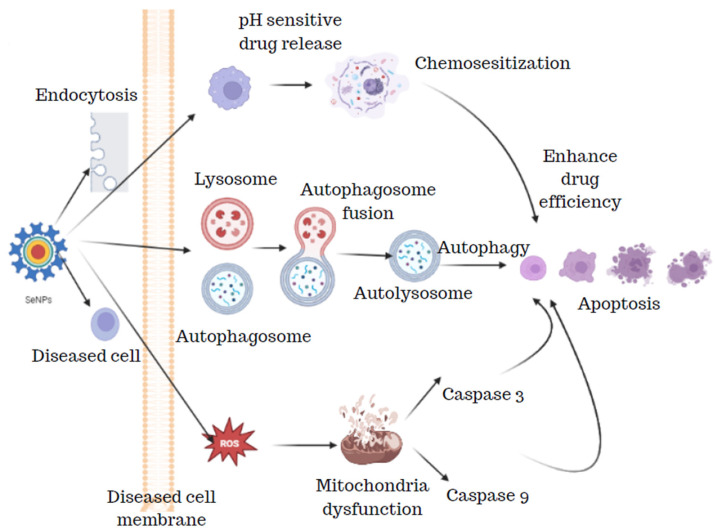
Scolicidal effect of selenium nanoparticles.

The SeNPs from the bacterial strain *Bacillus* sp. Msh-1 were studied for the scolicidal effect against hydatid cysts protoscoleces. Four different concentrations of SeNPs (50, 125, 250, and 500 µg/mL) were exposed to 0.5 mL of protoscoleces solution and 0.5 mL of SeNPs which were then kept at 37 °C for 10 min, 20 min, 30 min, and 60 min. After the incubation period was over, the upper portion was discarded and the protoscoleces were treated with 0.1% eosin stain which was viewed under the microscope and the dead protoscoleces were determined with normal saline as control. The results of these findings revealed that SeNPs showed potent scolicidal action at a concentration of 250 and 500 µg/mL at 10- and 20-min applications [52]. SeNPs have been known to have a pronounced scolicidal effect, where the SeNPs act on the diseased cell resulting in apoptosis by the release of ROS, lysosomes, autophagosomes, and also by the release of pH-sensitive drug release (Figure 10).

### 3.6. Wound-Healing Activity of Selenium Nanoparticles

Swiss albino rats were employed for this study by the authors, and 5 g of cream base was mixed with selenium nanoparticles of several concentrations. Rats were divided into four groups, the first group as control, group 2 was provided with an antibiotic (Lyramycin), group 3 with 5% of SeNPs, and group 4 with 10% of SeNPs. The dorsal side of the rats was wounded and the ointments were applied regularly and dressed.

**Figure 11:**
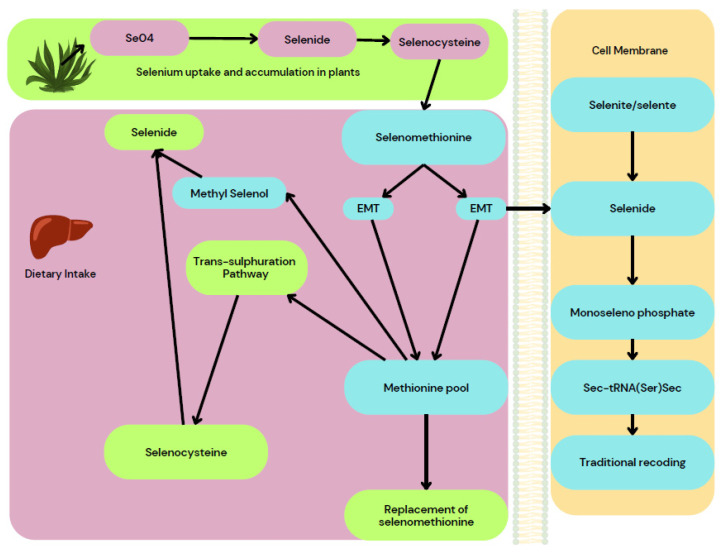
Wound-healing activity of Se nanoparticles.

The wound size was measured and photographed and the wound-healing percentage was calculated. The results revealed that a low dose of SeNPs healed the artificial wounds in 21 days and a high dose in 18 days, whereas the antibiotic took 21 days and it took 30 days for the wound to heal in control groups. Also, rats that were treated with high doses of SeNPs showed likeness to normal skin and normal hair growth on the affected area. Thus, the authors report that SeNPs appear to be more efficient in wound healing than Lyramycin antibiotic [50]. The wound-healing property of SeNPs acts on the trans-sulphuration pathway where the selenide and selenocysteine are converted to methionine and it will result in the replacement of selenomethionine that provides necessary action on the wound-healing property of SeNPs as depicted in Figure 11.

### 3.7. Toxicity of Selenium Nanoparticles

Shakibaie et al. have reported the toxicity level of SeNPs in mice synthesized from *Bacillus* sp. MSh-1 in comparison to synthetic SeNPs. They studied the toxicity level and their results showed that SeNPs expressed no biochemical changes for 10 mg/kg, but changes in body weight and hematological parameters were observed at 20 mg/kg, whereas selenium dioxide produced a high death rate at 2.5 mg kg^−1^ concentration inducing subacute toxicity in mice. Thus, they have given evidence for the toxic concentration of SeNPs, which can be applied to other pharmaceutical studies [38]. As SeNPs are known to have effective anticancer properties and can be employed as a chemo-preventive agent, understanding the toxicity level of selenium is mandatory to employ SeNPs for human use. Here, the sub-chronic toxic level of SeNPs was studied by Wang et al. The authors have reported the toxic nature of SeNPs by comparing it with selenite and high-Se protein. As stated by the authors, no reduction in hematological parameters was observed and no increase in ALT, AST, TP, and ALB-liver parameters was noticed. Also, the growth retardation and mottled liver surface were significantly less in comparison to selenite and high-Se protein. Therefore, SeNPs hold promise as an effective anticancer agent with a very less toxic effect [27].

**Figure 12:**
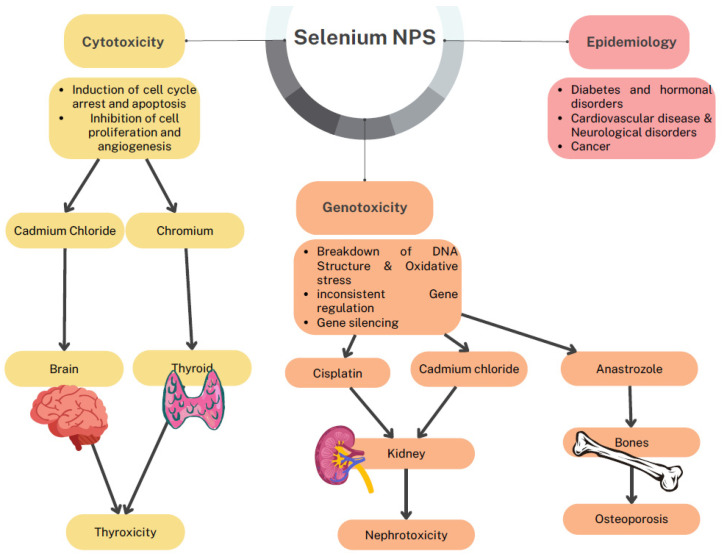
Toxicity of selenium nanoparticles.

To employ the SeNPs as an effective antimicrobial as well as antibiofilm agent, Khiralla and Deep have studied the toxicity nature of their tested concentration on Artemia larvae that produced no toxicity [48]. Figure 12 relates the different toxicity of SeNPs such as cytotoxicity, genotoxicity, and epidemiology. SeNPs have cytotoxic effects on the induction of cell cycle arrest and apoptosis. It also pronounces that effect on cell proliferation and angiogenesis. Genotoxicity of SeNPs results in the breakdown of DNA structure and oxidative stress; gene silencing and gene regulation are the important parameters of genotoxicity. SeNPs toxicity on different organs such as the thyroid is known as thyrotoxicity; in the kidney, it is known as nephrotoxicity, and when it affects bones, it results in osteoporosis. Acute toxicity of Se and SeNPs on *P. hypophthalmus* was analyzed at different concentrations where they confirmed from their study that SeNPs were found to be more toxic than Se at a 96 h treatment at a low dose of 3.97 mg/L. Also, the measurement of antioxidative stress indicated oxidative stress levels raised in organs like the gill, liver, and brain at an increased concentration of Se 4.5–6.0 mg/L and SeNPs at 2.5–4.0 mg/L. This indicated the toxic level of Se and SeNPs that could lead to the production of ROS due to their bio-accumulation in the organs. The neurotransmitter enzymes detected in terms of AChE activities in the liver, brain, and muscle were pointedly inhibited in Se and SeNPs. The levels of protein enzymes like AST, ALT, and carbohydrate metabolic enzymes like LDH and MDH activities with cortisol and HSP 70 of Se and SeNPs were significantly elevated. The histopathology study report of the liver and gill of *P. hypophthalmus* showed several deformities on introduction to Se and SeNPs. Thus, they determined that the toxicity of Se and SeNPs purely depends on the concentration and the quantity. It was found to be toxic at a higher concentration; also, SeNPs were reported to be more toxic than Se. Therefore, a low dose of Se is recommended as it is a vital micronutrient for humans and animals [49].

### 3.8. Anticancer Activity of Selenium Nanoparticles

**Figure 13:**
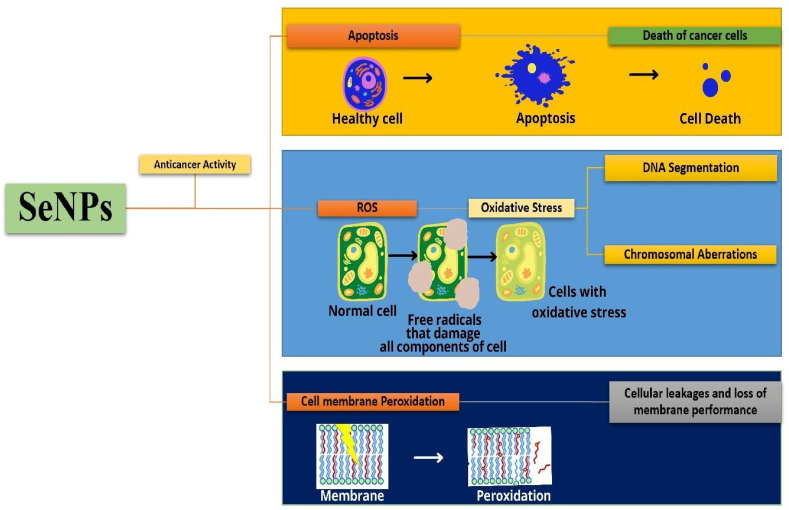
Mechanism of apoptosis and cell membrane peroxidation using SeNPs on cancer cells.

SeNPs have also demonstrated exceptional anticancer activity and showed great promise as cancer treatment and medication carriers. Other selenium compounds have lower antitumor activity than SeNPs. SeNPs’ anticancer effects are mediated by their capacity to stop cancer cells from growing by inducing cell cycle arrest in the S phase [60]. Beheshti et al. have reported the action of biogenically synthesized SeNPs against Leishmania major. Initially, they carried out in vitro studies using MTT and apoptosis assays where these SeNPs showed the highest cytotoxicity after 72 h and the IC_50_ value was at 1.62 ± 0.6 and 4.4 ± 0.6 µg/mL on promastigote and amastigote forms of leishmaniasis, respectively, whereas an apoptosis study showed that SeNPs exhibited DNA fragmentation in a promastigotes form. They also attempted to study the therapeutic effects of SeNPs in BALB/c mice by inducing cutaneous leishmaniasis. Their results conclude that selenium nanoparticles reduced the localized lesions after 14 days and also delayed the development of these lesions. Thus, these selenium nanoparticles can be employed for the treatment of lesions, ideally for cutaneous leishmaniasis [45]. Tianfeng et al. first reported the anti-proliferative effect of SeNPs synthesized from *U. pinnatifida* polysaccharide against A375 human melanoma cells. Their results showed that these SeNPs induce apoptotic cell death in A375 cells by inducing oxidative stress and mitochondrial dysfunction. Thus, this study gives a pathway to evaluate the chemotherapeutic properties of SeNPs against melanoma cancer [14]. Hector and related authors worked on a comparative study between chitosan SeNPs and other seleno species on the hepatocarcinoma cells to understand cell viability, proliferation, migration, and cell cycle. Results showed that Ch-SeNPs are an apt candidate for impaired tumor progression, as other selenospecies do not show much difference than untreated cells, and SeCyss2 caused high toxicity that might in turn affect the healthy cells. Thus, their study confirmed that chitosan-modified SeNPs are a potential applicant in cancer therapy (Figure 13) [12].

The mechanism of apoptosis and cell membrane peroxidation using SeNPs on the cancer cells is shown in Figure 13. The SeNPs cause apoptosis of the cancer cells leading to cell death. Another means of killing cancer cells is the release of reactive oxygen species that damage the components of the cell utilizing the release of free radicals. Cells will develop oxidative stress that results in DNA fragmentation and chromosomal aberrations. Peroxidation of cell membranes leads to membrane cleavage that leads to cellular leakage and loss of membrane performance. Huang et al. have synthesized Tf-SeNPs to investigate the cellular uptake of doxorubicin in cancer cells and they found that dox-loaded Tf-SeNPs exhibited cytotoxicity on MCF-7 cancer cell lines. The synthesized Tf-SeNPs activate ROS production, promoting apoptotic cell death. They also analyzed the activity of Tf-SeNPs, by inducing in vivo tumor development in a mice model. It was reported that they reduce tumor growth by the induction of p53-mediated apoptosis. Thus, these SeNPs are effective in treating cancer with efficacy and lesser side effects [24]. The SeNPs synthesized from the spirulina polysaccharides with a particle size of 48.6 nm were employed for the study on MCF-7, A375, MG-63, and HeLa-229 cancer cells. The in vitro anticancer analysis on the cancer cells exhibited a very low cytotoxic effect on the MCF-7, HeLa-229, and MG-63 cancer cells, whereas 50% cytotoxicity was observed for the A375 melanoma cancer cells. The mechanism behind the induction of apoptosis in these cells was studied through flow cytometry and DAPI staining. Thus, this study shows that SeNPs act as an effective anticancer drug against human cancer [46].

Zheng et al. have reported the anticancer effect of PEG-SeNPs on hepatocellular carcinoma that was synthesized from gray selenium. Initially, the human hepatoma cell line was cultured and tested for cell sensitivity to make it into a drug-resilient human hepatoma cell line (R-HepG2). Successfully, the authors have cultured R-HepG2 cells that showed 50-, 3.3-, and 2.5-fold high resilience to DOX, cisplatin, and Taxol, respectively. PEG-SeNPs with 6-coumarin were analyzed for their cellular uptake in HepG2 and R-HepG2 which was detected using a fluorescence probe. The results of the study indicate that the mechanism behind cellular uptake is endocytosis in the cancer cells. The results of MTT assay exhibited an enhanced inhibitory effect on R-HepG2 cells than on HepG2 cells, and morphological observation showed dose-dependent decrease in cell count and cell shrinkage. An investigation of molecular mechanisms exposed the depletion of mitochondrial membrane potential that induced apoptotic cell death on R-HepG2 cell lines. Thus, the authors conclude by stating the point that by using it as a surface decorator, PEG enhances the efficacy of SeNPs as an anticancer material against drug-resistant liver cancer [28]. Wadhwani et al. conducted a comparison study on the anticancer activity against breast cancer cells between chemically synthesized and biologically synthesized SeNPs. The authors have synthesized selenium nanoparticles from *Acinetobacter* sp. SW30 and employed these for the cancer study where they found that CSeNPs showed a pronounced effect on both NIH/3T3,4T1 and also MCF-7 cells than BSeNPs, but on scrutinizing the study further, CSeNPs showed higher cytotoxicity than BSeNPs. Thus, the authors conclude that BSeNPs can be employed for anticancer activity as CSeNPs but can cause toxic effects on cells. Thus, BSeNPs act as a good alternative to anticancer agents [47]. Kong et al. have studied the mechanism of SeNPs that suppresses the growth of prostate cancer cells (LNCaP). The synthesized SeNPs were of 10–30 nm in size which strongly suppressed the prostate LNCaP cancer cells partially through caspases-mediated apoptosis. Their mechanistic study on nano-Se confirmed that it inhibits the growth of prostate cancer cells through AR suppression, which is necessary for both androgen-dependent and independent prostate cancers. Also, it was found that downregulation of AR induced rapid degradation of it by the SeNPs. From their results, it was also known that SeNPs activated the Akt/Mdm2 pathway initiating AR phosphorylation, ubiquitination, and finally degradation. Thus, the authors conclude that suppression of prostate cancer cells occurred by regulation of AR transcription and promotion of AR protein degradation. As SeNPs disrupt the androgen receptor, they act as a potential agent in cancer treatment [30].

Li et al. synthesized Se-capped MUN functionalized nanoparticles which were investigated for their protective effect against cisplatin-induced nephrotoxicity on HK-2 tubular cells. The mechanism behind this was studied by the authors that Se-capped MUN blocked caspase-mediated apoptosis that was persuaded by cisplatin by inhibiting the overproduction of ROS. Thus, their study confirmed that promising effect of e species in the prevention of cisplatin-induced renal injury [31]. Se-capped Trolox have improved antioxidant activity and blocked the cisplatin-induced cell growth on HK-2 cells. This was confirmed by their study on DNA fragmentation, chromatic condensation, PRAP cleavage, and activation of caspase-3. Thus, their study confirmed that Se-capped Trolox acts as a potential species that has potential application in the injury induced by cisplatin [32]. Functionalization of a chemotherapeutic drug 5-fluorouracil with SeNPs was prepared and employed as an anticancer agent. Several human cell lines were used in their study which includes MCF-7 breast adenocarcinoma cells, HepG2 hepatocellular carcinoma cells, A375 melanoma cells, Colo201 colon adenocarcinoma cells, and PC-3 prostatic carcinoma cells which were susceptible to 5FU-SeNPs; also, it was able to select between cancer and normal cells. The apoptotic induction of this Se-functionalized 5-FU in human melanoma cells was studied through various in vitro techniques like DNA fragmentation assay and nuclear condensation. They also conducted an in vivo study where the results indicated that 5FU-SeNPs act as a potential anticancer agent with very low Se toxicity. Altogether, they provide a way to employ SeNPs as a carrier of 5FU to achieve anticancer activity [33]. Selenium nanoparticles synthesized using fresh lemon leaves were studied for the UV-induced DNA injury. Results of their study depicted a reduction in the genetic damage of human lymphocytes caused due to UVB by the synthesized selenium nanoparticles [38]. MCF-7 cell lines treated with SeNPs at different concentrations were prepared using biological and chemical methods. Results of their study showed no difference in the activity of SeNPs synthetized using different methods; however, a significant increase in the release of LDH and decrease in the cell viability were observed at 36 h and 6 h at lowest concentration of 25 µg.mL and highest concentration of 100 µg.mL, respectively. Also, doxorubicin showed less than 20% cell death at 24 h, whereas a combined effect of SeNPs and doxorubicin produced more than 50% cell death at 24 h, whereas the combination of selenious acid and doxorubicin and fenugreek seeds did not show any cytotoxicity. Thus, the authors confirm that the conjugated effect of selenium nanocomposites facilitated the cellular uptake of doxorubicin to induce cytotoxicity on MCF-7 cells through apoptosis [49]. Ramya et al. have studied the cytotoxicity potential of SeNPs synthesized from an actinobacterium sp. on hepatic carcinoma and cervical carcinoma where the cells were cultured and tested using MTT assay. The results of this study showed that 99.5% of cell death in HepG2 cells was observed at 50 µg concentration whereas at 100 µg concentration 100% of cell death was observed for HeLa cervical cancer cell lines. Hence, the authors conclude that *Streptomyces minutiscleroticus*-synthesized selenium nanoparticles displayed inhibition of high cell growth for both HeLa and HepG2 cell lines [53].

## 4. Conclusions and Future Perspectives

Selenium is an essential element that provides nutritional value to carry out all physiological processes and an adequate supply of selenium is important to avoid selenium deficiency health defects. Nano-selenium is the best form to regulate supplementation as it has low toxicity, is easily available, and is adsorbed by the system. SeNPs can be easily synthesized through various agents like plants, bacteria, viruses, fish, etc. SeNPs play a role in the dietary supplement and also act as an anti-viral, antioxidant, antibiofilm, antibacterial, anticancer, and scolicidal agent. Many studies on toxicity have revealed that SeNPs are less toxic at the appropriate dosage. However, with further studies on experimental models, Se nanoparticles will play a role in the healthcare industry.

## Figures and Tables

**Table 1:** Literature report on the physical synthesis of selenium nanoparticles.

S. No.	Method	Employed Method	Application	Reference
1		Excimer pulsed laser ablation	-	[17]
2	Synthesis of selenium	Femtosecond pulsed laser ablation	Antibiofilm agent	[18]
3	nanoparticles by physical	Femtosecond laser-induced plasma shock wave	-	[19]
4	method	Microwave irradiation	Solar cell	[20]
5		Pulsed laser ablation	-	[21]
6		Laser irradiation	-	[22]

**Table 2:** Literature report on the selenium nanoparticles that were synthesized using chemical method.

S. No.	Chemicals Employed for SeNP Synthesis	Application	Reference
1	2,2′-azo-bis-(2-amidnopropane) hydrochloride (AAPH), 1,1-diphenyl-2-picryhydrazyl (DPPH), selenium dioxide (Na_2_SeO_3_)	Free radical scavenging activity	[23]
2	selenium dioxide (Na_2_SeO_3_), transferrin (Tf), 1-(3-Dimethylaminopropyl)-3-ethylcarbodiimide hydrochloride (EDC), chitosan (CS)	Cancer	[24]
3	quercetin (Qu), acetylcholine (Ach) to the surface of Se nanoparticles (Qu–Ach@SeNPs)	Antibacterial activity	[25]
4	sodium selenite, glutathione bovine serum albumin	Inhibits bacterial growth	[26]
5	epigallocatechin-3-gallate, sodium selenite	Toxicity	[27]
6	polyethylene glycol, Se powder	Anticancer activity on hepatocellular carcinoma	[28]
7	selenium nanoparticle (3–20 nm)	Protective ability of selenium NP against thyrotoxicity	[29]
8	sodium selenite, glutathione bovine serum albumin	Prostate anticancer activity	[30]
9	sodium selenite, 11-mercapto-1-undecanol	Reversal of nephrotoxicity	[31]
10	Trolox, sodium selenite	Prevention of cisplatin-induced renal injury	[32]
11	5-fluorouracil, sodium selenite	Anticancer synergism	[33]
12	chitosan, sodium selenite	Antioxidant capacity	[34]
13	sodium alginate, sodium selenite	Anticancer activity	[35]

**Table 3:** Literature survey on various biological methods of the synthesis of selenium nanoparticles.

S. No	Biological Source Used	Application	Reference
1	TGA Virus	Immunogenic properties	[43]
2	Glucose	Biological application	[11]
3	*Bacillus cereus*	Biological application	[10]
4	*Bacillus* sp. MSh-1	Acute and sub-acute toxicity	[44]
5	*Bacillus* sp. MSh-1	Treatment of leishmaniasis	[45]
6	Polysaccharide of *Untaria pinnatifida* algae	Human melanoma cells	[14]
7	Chitosan	Hepatocarcinoma (HepG2) cells	[12]
8	*Spirulina* Polysaccharides	Anticancer activity on A375 human melanoma cells, breast cancer cells (4T1, MCF-7)	[46]
9	*Acinetobacter* sp. SW30	Antimicrobial activity	[47]
10	*Bacillus licheniformis*	Antimicrobial activity	[48]
11	*Labeo rohita*	Toxicity study	[49]
12	L-cysteine	-	[42]
13	*Vitis vinifera*	-	[50]
14	*Aspergillus terreus*	-	[51]
15	*Bacillus* sp. Msh-1	Treatment of cystic echinococcosis	[52]
16	Lemon leaves	Protective effect on UV-induced DNA damage	[38]
17	Fenugreek seeds	Cytotoxicity on human breast cancer cells	[39]
18	*Streptomyces minutiscleroticus*	Antibiofilm, antioxidant, anti-proliferative, wound healing activity against HeLa	[53]
19	*Bacillus* sp. Msh-1	Antibiofilm activity	[54]
20	*Zooglea ramigera*	-	[40]

## Data Availability

No new data were created or analyzed in this study. Data sharing is not applicable to this article.
